# SARS-CoV-2 infects olfactory neurons and basal stem cells and induces axonal degeneration through TRPV1 activation

**DOI:** 10.1016/j.isci.2026.116098

**Published:** 2026-05-25

**Authors:** Vanessa Anna Co, Siwen Liu, Rachel Chun-Yee Tam, Bobo Wing-Yee Mok, Alvin Hiu-Chung Lam, Honglin Chen, Yiling Hong

**Affiliations:** 1 Centre for Virology, Vaccinology and Therapeutics, The University of Hong Kong, Hong Kong SAR, P.R. China; 2 State Key Laboratory for Emerging Infectious Diseases & Department of Microbiology, Li Ka Shing Faculty of Medicine, The University of Hong Kong, Hong Kong SAR, P.R. China; 3 College of Veterinary Medicine, Western University of Health Sciences, Pomona, CA 91766-1854, USA

**Keywords:** Molecular biology, Neuroscience, Cell biology, Cell

## Abstract

Neurological complications such as anosmia are among the most frequent and persistent symptoms of COVID-19; yet, the mechanisms linking SARS-CoV-2 infection to sensory neuronal injury remain unclear. We demonstrate that SARS-CoV-2 directly infects olfactory sensory neurons through ACE2 upregulation and stimulates basal stem cell proliferation, as shown in human iPSC-derived sensory neurons and validated in golden Syrian hamsters. Exposure to live viruses or its S1 spike protein induces TRPV1 channel redistribution from the nucleus to the plasma membrane, resulting in axonal degeneration. Single-nucleus RNA sequencing reveals activation of exocytosis and transmembrane transport pathways with disruption of axonal guidance networks. Pharmacological inhibition of TRPV1 with capsazepine mitigates neuronal injury and preserves axonal integrity. These findings identify TRPV1 activation as a central mediator of SARS-CoV-2-induced neurodegeneration in the olfactory epithelium and suggest that TRPV1 antagonism offers a promising therapeutic avenue for treating COVID-19-related anosmia.

## Highlights

• BA.5 virus infects olfactory neurons and basal stem cells in iPSC and hamster models
• BA.5 virus or S protein increases ACE2 and drives TRPV1 redistribution causing axonal loss
• TRPV1 inhibitor capsazepine reduces channel activation and axonal injury in sensory neurons

## Graphical abstract

**Figure:**
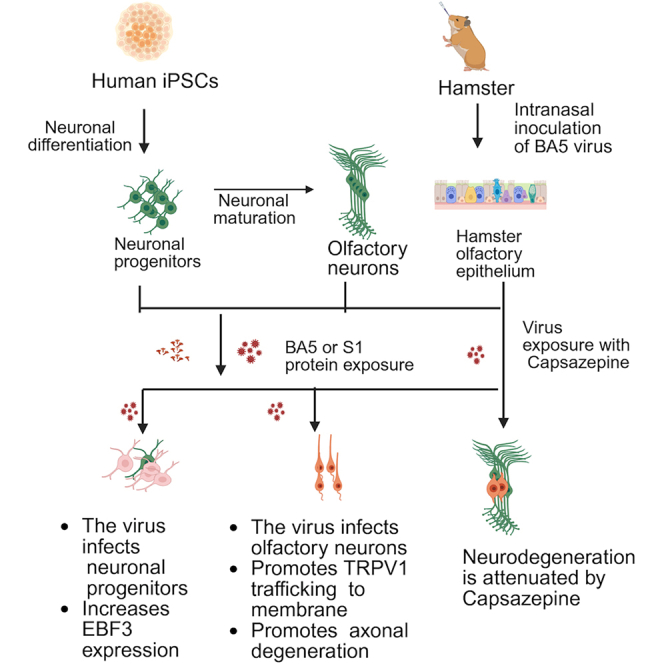

## Introduction

The emergence of severe acute respiratory syndrome coronavirus 2 (SARS-CoV-2) caused the global coronavirus disease 2019 (COVID-19) pandemic.^1^ Beyond its well-recognized pulmonary pathology, SARS-CoV-2 infection frequently manifests with neurological symptoms, including anosmia, headache, and cognitive impairment.^2^^,^^3^ Postmortem analyses have revealed widespread viral dissemination across both respiratory and non-respiratory tissues, including the central nervous system,^4^ and viral RNA and proteins have been detected in the olfactory cortex of non-human primates.^5^ The hallmark symptoms of anosmia and ageusia^6^ underscore a direct clinical association between SARS-CoV-2 infection and olfactory dysfunction.

The olfactory nerve originates in the olfactory epithelium (OE), whose sensory axons traverse the cribriform plate to reach the olfactory bulb, where odor information is processed and transmitted to higher cortical centers.^7^ Unlike most cranial nerves, the olfactory system retains robust regenerative capacity throughout life, making it an ideal model for studying neuronal repair and regeneration. New olfactory sensory neurons (OSNs) arise from basal stem cells that replace aged or damaged neurons, maintaining epithelial homeostasis.^8^ Two principal basal progenitor populations—horizontal basal cells (HBCs) and globose basal cells (GBCs)—drive this process: HBCs serve as a quiescent stem-cell reservoir activated by injury, subsequently differentiating into GBCs, which proliferate and give rise to neurons and supporting epithelial cells.^9^ Because the OE directly interfaces with the external environment, it serves as a primary site of viral entry.^10^ Damage to olfactory axons and dendrites contributes not only to smell loss but also to impaired higher cognitive processing.^11^ Clinical imaging studies further demonstrate a correlation between anosmia severity and reduced OE thickness.^12^

Recent studies have shown that most HBCs express angiotensin-converting enzyme 2 (ACE2) and transmembrane protease serine 2 (TMPRSS2), the key viral entry factors for SARS-CoV-2.^10^ In murine models, SARS-CoV-2 nucleocapsid (N) protein has been detected within HBCs, suggesting that infection may impair their regenerative capacity and contribute to long-term anosmia.^10^ However, the degree to which sensory neurons themselves are susceptible remains controversial. Some studies report limited neuronal infection due to low ACE2 expression, implying that neuropathology may stem from indirect inflammatory or immune responses.^10^^,^^13^ In contrast, other investigations have detected ACE2 in olfactory and other sensory neurons, supporting the possibility of direct viral infection.^14^^,^^15^

Transient receptor potential vanilloid 1 (TRPV1) is a calcium-permeable, non-selective cation channel abundantly expressed in primary sensory neurons with unmyelinated C fibers located in the dorsal root and trigeminal ganglia.^16^ TRPV1 participates in multiple physiological and developmental processes, including intracellular Ca^2+^ homeostasis, axonal guidance, neurite extension, cell proliferation and differentiation, chemotaxis, and immune regulation.^17^^,^^18^ Functionally, TRPV1 acts as a molecular “attack sensor” that detects harmful physical or chemical stimuli under inflammatory conditions.^19^ Channel activation and stability are modulated by phosphorylation and palmitoylation via protein kinases and palmitoyltransferases, facilitating its trafficking from intracellular stores to the plasma membrane where it becomes fully functional.^19^^,^^20^ Upregulation of TRPV1 and insertion to the membrane enhances nociceptive signaling and promotes neuropeptide release, driving neurogenic inflammation.^16^ Moreover, TRPV1 activation disrupts cytoskeletal organization by disassembling microtubules, thereby impairing axonal growth, morphology, and neuronal migration.^21^ Prolonged activation results in excessive Ca^2+^ influx, depolarization, and neuronal death.^22^^,^^23^^,^^24^ Aberrant TRPV1 signaling has been implicated in numerous neurodegenerative and neuroinflammatory disorders.^25^ Notably, TRPV1 expression is upregulated during infection by several respiratory and systemic viruses—including human rhinovirus, respiratory syncytial virus, measles virus, and hepatitis C virus—suggesting a conserved role in virus-induced neuroimmune activation.^26^ Defining the contribution of TRPV1 to SARS-CoV-2-mediated neuronal injury within the upper airway and olfactory system is therefore of high mechanistic and clinical importance.

To investigate the interaction between SARS-CoV-2 and sensory neurons, we utilized human induced pluripotent stem cell (iPSC)-derived sensory neurons and golden Syrian hamster models. Neurons were either infected with live SARS-CoV-2 or exposed to the spike (S1) subunit, which mediates viral attachment and fusion with host membranes.^27^^,^^28^ The S1 subunit has been shown to activate airway sensory C fibers.^29^ We observed that approximately 5% of sensory neurons—predominantly olfactory neurons—were directly infected by SARS-CoV-2. Furthermore, the virus infected immature OSNs and basal stem cells, accompanied by increased expression of Early B cell Factor 3 (EBF3). EBF3 is a neurodevelopmental transcription factor whose mutations cause hypotonia, ataxia, and delayed development syndrome.^30^^,^^31^ Loss of EBF3 disrupts olfactory receptor neuron innervation of the olfactory bulb in mice,^32^ whereas EBF3 overexpression promotes neuronal differentiation in *Xenopus* embryos.^33^ Following viral or S1 exposure, TRPV1^+^ sensory neurons exhibited upregulated TRPV1 expression, cytosolic redistribution, and prominent insertion into the plasma membrane, accompanied by altered axonal extension patterns.

## Results

### Exposure to omicron BA.5 virus and its spike protein S1 subunit upregulated ACE2 expression in iPSC-derived sensory neurons

**Figure 1:**
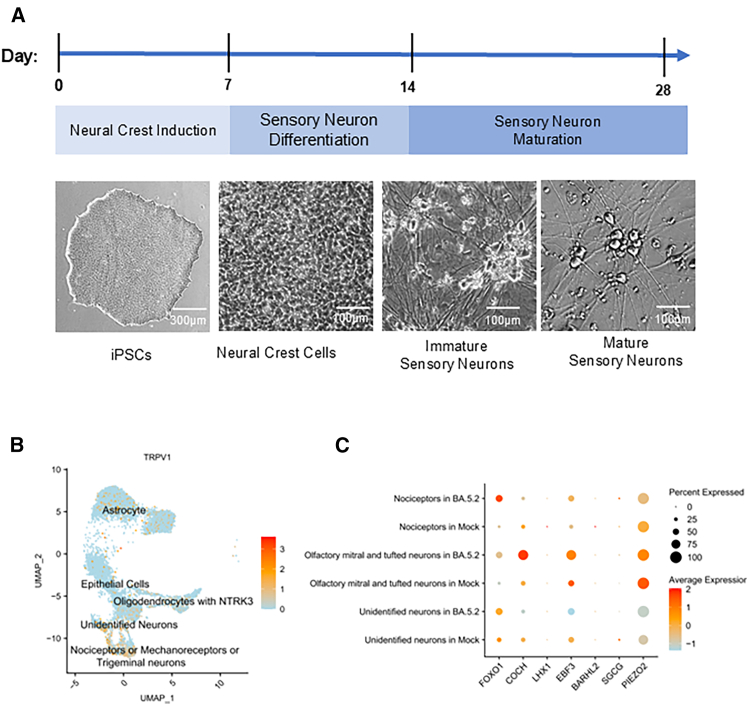
Generation and characterization of iPSC-derived sensory neurons (A) Schematic diagram and phase-contrast images illustrating the differentiation of human iPSCs into sensory neurons via a neural crest intermediate. Phase-contrast images were captured using an EVOS M100 fluorescence microscope with a 10× objective. Scale bars, 300 μm or 100 μm as indicated. (B) UMAP projection of snRNA-seq data showing cell-type heterogeneity in the differentiated neuronal population, including nociceptors, mechanoreceptors, trigeminal neurons, undefined neurons, astrocytes, oligodendrocytes, and epithelial cells. TRPV1 expression levels are displayed across neuronal subtypes. (C) Dot plot of snRNA-seq data showing differential gene expression in mitral and tufted cell clusters following viral exposure.

Peripheral sensory neurons were differentiated from neural crest cells derived from human iPSCs cultured *in vitro*. These neural crest cells were subsequently maintained in supplemented differentiation medium to promote their maturation into mature peripheral sensory neurons (Figure 1A). Single-nucleus RNA sequencing (snRNA-seq) coupled with uniform manifold approximation and projection (UMAP) analysis revealed pronounced cellular heterogeneity within this differentiation platform, identifying clusters corresponding to nociceptors, mechanoreceptors, trigeminal neurons, undefined neurons, astrocytes, oligodendrocytes, and epithelial cells. TRPV1 expression was highly enriched in nociceptors, mechanoreceptors, and trigeminal neurons (Figure 1B).

When these iPSC-derived sensory neurons were exposed to the SARS-CoV-2 Omicron BA.5 variant—originally isolated from COVID-19 patients in Hong Kong (hCoV-19/Hong Kong/HKU-220712-005/2022)—at a multiplicity of infection (MOI) of 1.0, RNA-seq dot-plot analysis revealed significant transcriptional alterations in neuronal subtypes, including nociceptors, mitral cells, and tufted neurons, with differential expression of genes such as FOXO1, COCH, EBF3, and PIEZO2 (Figure 1C). Among these, EBF3 expression was markedly upregulated. EBF3 is a transcription factor highly expressed in olfactory neurons and HBCs of the OE, where it plays a critical role in regulating neuronal differentiation and development.

**Figure 2:**
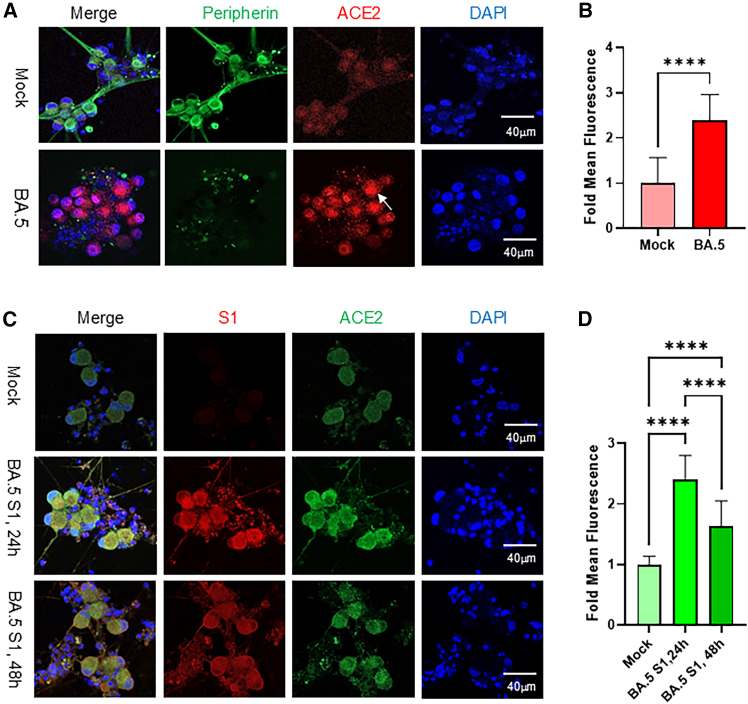
Exposure to live Omicron BA.5 virus or its spike protein S1 subunit upregulates ACE2 expression in iPSC derived sensory neurons (A) Immunostaining of mature sensory neurons exposed to Omicron BA.5 virus (MOI 1.0) or mock-exposed for 48 h. Cells were fixed and stained for peripherin (green) and ACE2 (red), with DAPI marking nuclei. Virus exposure induced ACE2 upregulation, neuronal soma enlargement, and loss of peripherin-positive processes. Images were acquired using a confocal microscope with a 20× objective. Scale bars, 50 μm. (B) ACE2 fluorescence intensity was quantified in five neurons per field across three independent experiments using ImageJ and normalized to mock controls. Data are shown as mean ± SEM, analyzed by student’s *t* test using GraphPad Prism 10. ∗∗∗∗*p* < 0.0001. (C) ACE2 expression following exposure to the BA.5 S1 spike protein subunit (100 ng mL^−1^) for 24 or 48 h. Neurons were immunostained for S1 (red), ACE2 (green), and DAPI (blue). Images were captured with a 40× objective. Scale bars, 20 μm. (D) ACE2 fluorescence intensity was quantified in five neurons per field across three independent fields using ImageJ and normalized to mock controls. Data are shown as mean ± SEM, analyzed by student’s *t* test using GraphPad Prism 10. ∗∗∗∗*p* < 0.0001.

To assess viral effects on receptor expression, immunofluorescence staining was performed to detect peripherin—a peripheral neuron marker—and ACE2, the SARS-CoV-2 entry receptor. Under control conditions, most sensory neurons exhibited low but detectable ACE2 expression (red; upper images). Upon exposure to the Omicron BA.5 virus, ACE2 expression was significantly upregulated in a subset of neurons, predominantly within EBF3-positive cells (Figure S1). This upregulation was accompanied by enlargement of the neuronal soma and a marked reduction in peripherin staining within both neuronal soma and processes, indicative of neuronal stress and axonal injury (Figures 2A and 2B). In addition, immunostaining with the apoptotic marker caspase 3 together with the OSN marker OMP revealed that SARS-CoV-2 infection induced neuronal apoptosis (Figure S2).

To determine whether this ACE2 upregulation resulted directly from viral spike protein engagement, neuronal network cultures were exposed to the S1 subunit of the BA.5 spike protein. Following exposure, the cells were thoroughly washed with PBS, fixed, and analyzed for ACE2 expression at 24 and 48 h post-infection (hpi). The results showed that the SARS-CoV-2 S1 spike protein binds selectively to subsets of sensory neurons, triggering a pronounced upregulation of ACE2 (green) within 24 h of exposure (Figures 2C and 2D).

### Omicron BA.5 virus infected TRPV1-positive sensory neurons, including olfactory neurons and basal stem cells in cell culture and golden Syrian hamster models

**Figure 3:**
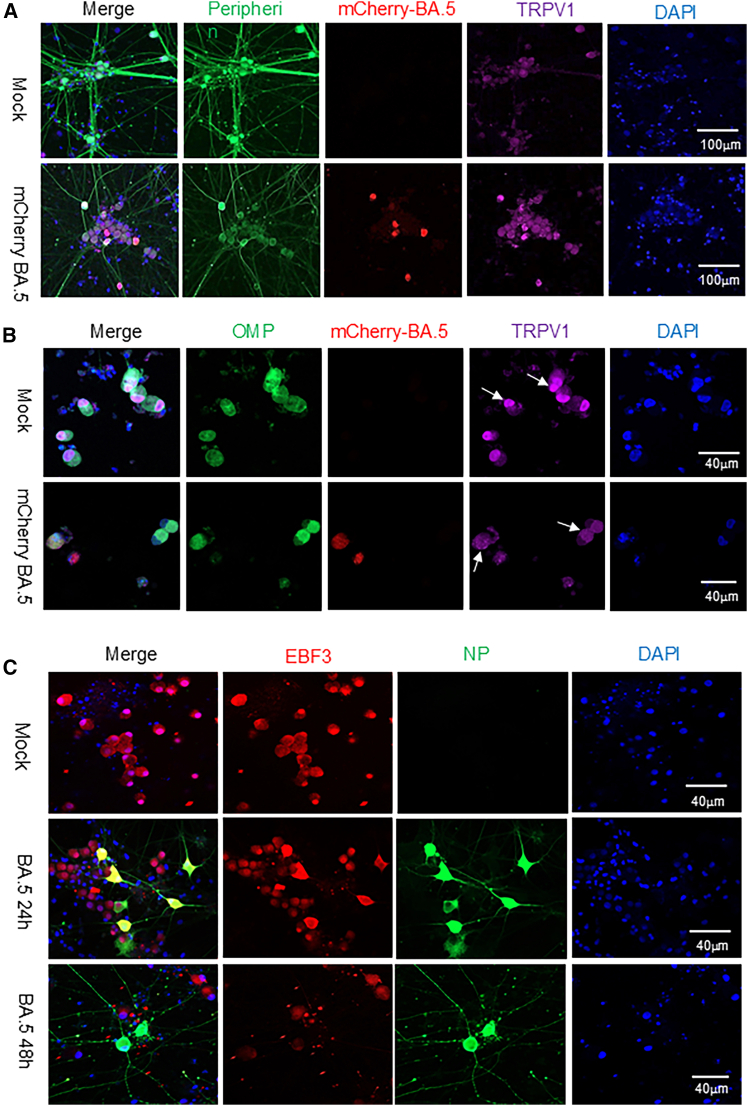
BA.5 virus directly infects TRPV1-positive olfactory neurons in human iPSC-derived cultures (A) Immunostaining sensory neurons 48 h post infection with mCherry-expressing BA.5 virus (red) or mock exposure. Peripherin (green) and TRPV1 (purple) identify neuronal populations; DAPI (blue) labels nuclei. Images were acquired with a confocal microscope at 10× magnification. Scale bars, 100 μm. (B) Co-immunostaining of infected neurons for mCherry, olfactory marker protein (OMP, green), and TRPV1 (purple) demonstrates viral infection in olfactory sensory neurons. Scale bars, 40 μm. (C) Immunostaining for SARS-CoV-2 BA.5 NP (green) and EBF3 (red) confirm infection within olfactory neurons. Images were acquired at 40× magnification. Scale bars, 40 μm.

To determine whether specific subtypes of sensory neurons can be directly infected by the BA.5 virus, we engineered a recombinant BA.5 strain expressing the fluorescent reporter mCherry by inserting the mCherry gene into the F6 fragment of the SARS-CoV-2 BA.5 genome using the circular polymerase extension reaction method.^34^ The mCherry-expressing BA.5 virus was applied to neuronal cultures at MOIs of 0.1, 1.0, and 2.0, followed by incubation at 37°C in 5% CO_2_. Neurons were monitored daily for mCherry fluorescence using a fluorescence microscope to identify infected cells. At 24 hpi, mCherry fluorescence was detected in the 1.0 and 2.0 MOI groups (preliminary data not shown), and by 48 hpi, fluorescence was observed in all infection groups. An MOI of 1.0 was selected for subsequent experiments based on consistent infection efficiency. Approximately 5% of TRPV1-positive sensory neurons were directly infected at 24 hpi (Figure 3A). Given that TRPV1 is strongly expressed in nociceptive, trigeminal, and olfactory neurons (Figure 1B), we next sought to identify which neuronal subtypes are most susceptible to SARS-CoV-2 infection.

Cell cultures exposed to the BA.5 variant were analyzed by immunofluorescence staining using antibodies against SARS-CoV-2 nucleocapsid protein (NP), olfactory marker protein (OMP), and the olfactory neuron transcription factor EBF3. The majority of NP-positive infected cells co-expressed OMP and EBF3, indicating that OSNs are a major target of SARS-CoV-2 infection *in vitro* (Figures 3B and 3C). Confocal imaging at 40× magnification revealed that infected cells exhibited morphological features characteristic of mitral cells, tufted cells, interneurons, and OSNs (Figure S3).

**Figure 4:**
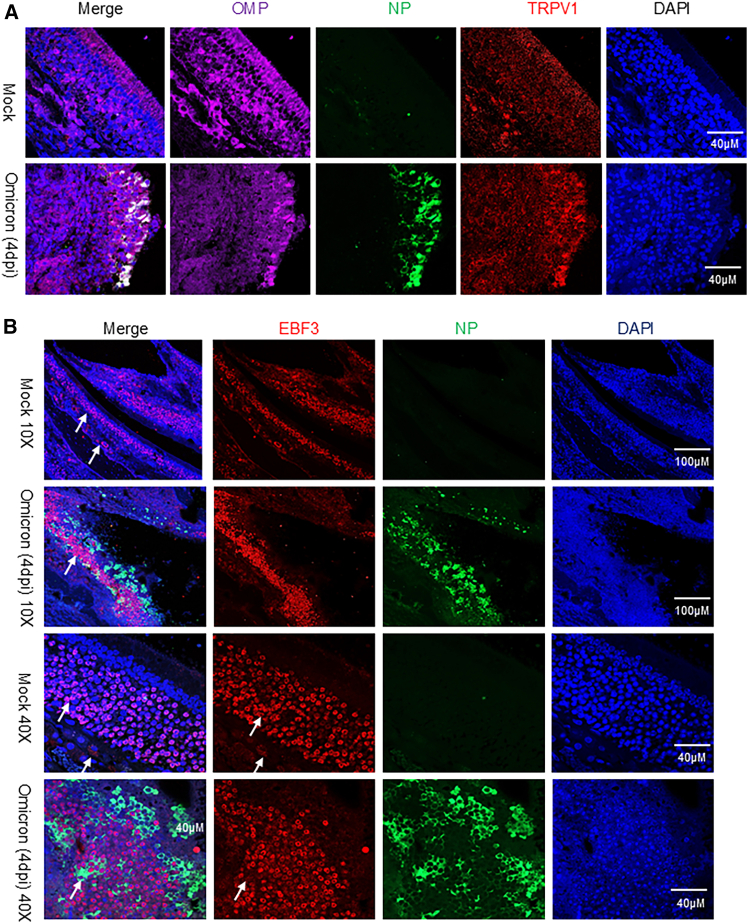
SARS-CoV-2 BA.5 infects the hamster OE and basal cells (A) Colocalization of SARS-CoV-2 NP (green) and TRPV1 (red) with the olfactory sensory neuron marker OMP (purple) in hamster OE indicates viral infection of mature olfactory sensory neurons. (B) Colocalization of NP (green) with EBF3 (red) in basal cells and immature olfactory neurons demonstrates infection of progenitor and developing neuronal populations within the hamster olfactory turbinate.

To corroborate these findings *in vivo*, we employed a golden Syrian hamster model. Four days after intranasal inoculation with SARS-CoV-2 BA.5, nasal turbinate tissues were subjected to double immunofluorescence staining with antibodies against SARS-CoV-2 NP, OMP, and EBF3. In Syrian hamsters, NP immunoreactivity was widespread throughout the OE, which exhibited extensive structural damage. NP expression colocalized with OMP-positive neurons, confirming that OSNs are highly susceptible to infection. Moreover, viral exposure markedly upregulated TRPV1 expression within the infected OE (Figure 4A).

NP was also detected in EBF3-expressing cells, most of which corresponded to immature OSNs and basal stem cells located beneath the mature neuronal layer. The number of EBF3-positive cells increased significantly following viral infection (Figures 4B and S4). These observations suggest that SARS-CoV-2 infection not only targets mature sensory neurons but also disrupts progenitor populations, thereby compromising the regenerative capacity of the OE.

### Omicron BA.5 virus and its spike protein S1 subunit induce TRPV1 translocation from the nucleus to the cytosol and plasma membrane

**Figure 5:**
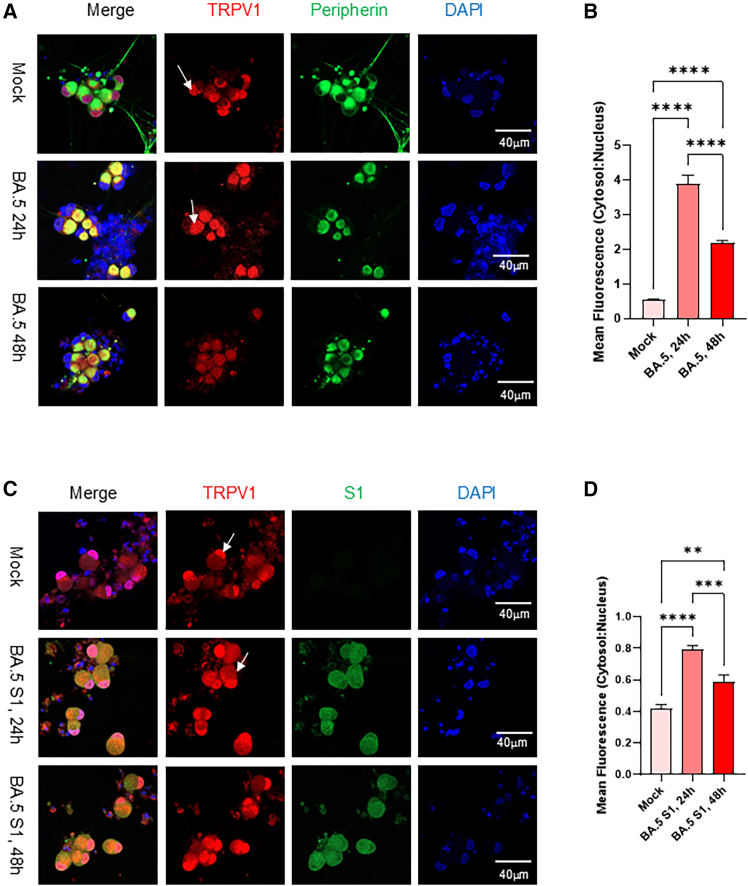
Effect of BA.5 virus and S1 spike protein subunits on TRPV1 expression and subcellular localization (A) Immunostaining of sensory neurons exposed to BA.5 virus or mock control for TRPV1 (red) and peripherin (green), with DAPI (blue) for nuclei. White arrows indicate TRPV1 localization. Virus exposure upregulated TRPV1 expression and induced translocation from the nucleus to the cytosol and plasma membrane. (B) TRPV1 fluorescence intensity and cytosol-to-nucleus subcellular distribution were quantified in five neurons per slide across three independent experiments following viral exposure. Data are shown as mean ± SEM, analyzed by student’s *t* test using GraphPad Prism 10. ∗∗∗∗*p* < 0.0001. (C) Sensory neurons exposed to BA.5 S1 protein (100 ng mL^−1^) or mock control were stained for S1 (green) and TRPV1 (red). Arrows indicate increased cytosolic TRPV1 localization in S1-treated neurons, similar to viral exposure. (D) TRPV1 fluorescence intensity and cytosol-to-nucleus subcellular distribution were quantified in five neurons per slide across three independent experiments following viral exposure. Data are shown as mean ± SEM, analyzed by student’s *t* test using GraphPad Prism 10. ∗∗*p* < 0.01, ∗∗∗∗*p* < 0.001, ∗∗∗∗*p* < 0.0001. Following S1 treatment.

TRPV1 can be activated by a variety of physical and chemical stimuli, including capsaicin, heat, low pH, and environmental toxins, triggering acute nociceptive pain and neurogenic inflammation. To assess TRPV1 activation in sensory neurons following viral exposure, we examined the effects of the SARS-CoV-2 Omicron BA.5 variant and its spike protein S1 subunit. Sensory neurons infected with BA.5 were analyzed by immunofluorescence staining using peripherin (green) and TRPV1 (red) antibodies at 24 and 48 hpi. Upon exposure to the virus, the neuronal soma became noticeably enlarged, and exhibited strong upregulation of TRPV1 expression. This increase was accompanied by a striking redistribution of TRPV1 from the nucleus to the cytosol and plasma membrane. In contrast, mock-treated control neurons displayed predominantly nuclear TRPV1 localization, where it was colocalized with DAPI (Figure 5A). Quantification of fluorescence intensity across cellular compartments confirmed a marked increase in cytosolic and membrane-associated TRPV1 following viral infection (Figure 5B).

Exposure of neuronal cultures to the purified S1 subunit of the BA.5 spike protein (100 ng mL^−1^) produced similar effects. Dual immunostaining for S1 (green) and TRPV1 (red) revealed extensive colocalization, appearing as yellow puncta, indicating that the viral S1 subunit directly interacts with TRPV1. The S1 subunit induced TRPV1 upregulation and promoted its translocation from the nucleus to the cytosol; however, a portion of TRPV1 remained within the nucleus after 48 h of exposure (Figures 5C and 5D).

**Figure 6:**
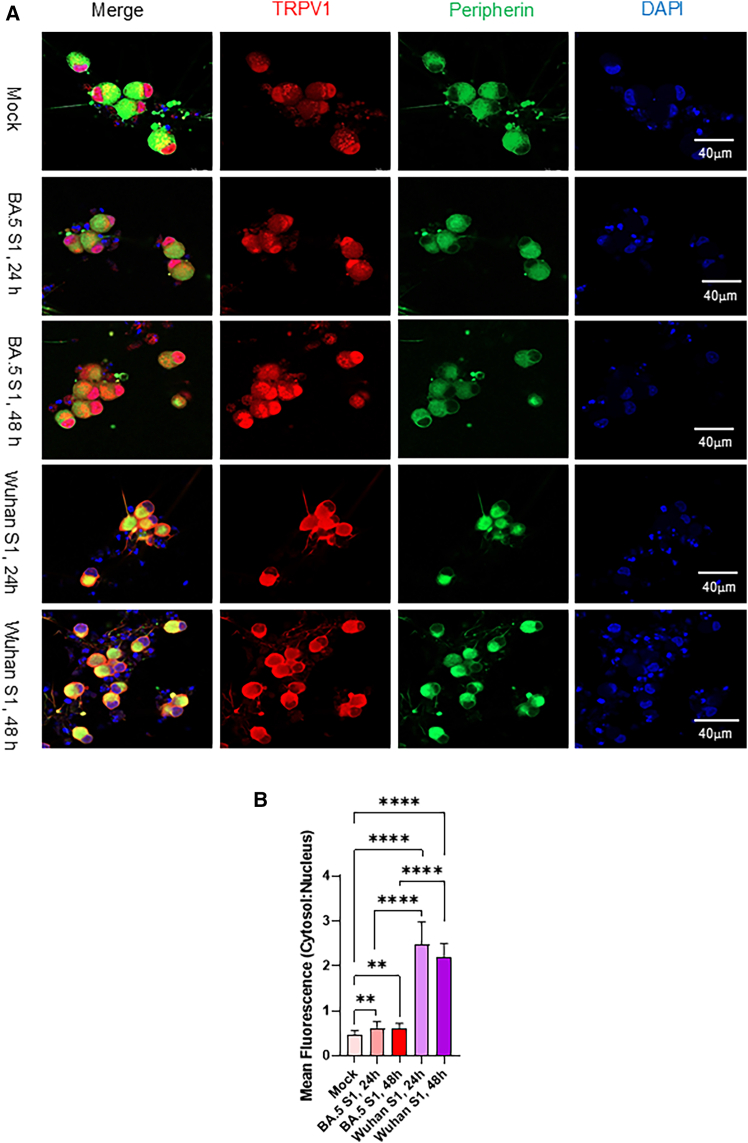
Effects of Wuhan S1 spike protein subunits on TRPV1 expression and subcellular trafficking (A) Comparison of TRPV1 localization in neurons exposed to SARS-CoV-2 Omicron BA.5 or ancestral Wuhan S1 spike protein subunits. Images were acquired using a confocal microscope with a 40× objective. Scale bars, 20 μm. (B) TRPV1 fluorescence intensity and cytosol-to-nucleus subcellular distribution were quantified in five neurons per slides across three independent experiments following BA.5 or Wuhan S1 proteins exposure. Data are presented as mean ± SEM and analyzed using student’s *t* test in GraphPad Prism 10. ∗∗∗∗*p* < 0.0001, ∗∗*p* < 0.01, ∗*p* < 0.05.

We next compared TRPV1 activation induced by the S1 subunits from the Omicron BA.5 and the ancestral Wuhan SARS-CoV-2 strains, the latter known for its higher pathogenicity. The Wuhan S1 protein elicited a more robust response, producing greater TRPV1 upregulation and enhanced translocation to the cytosol, accompanied by a corresponding reduction in nuclear TRPV1 (Figures 6A and 6B). Collectively, these findings indicate that exposure to the SARS-CoV-2 spike S1 protein—particularly from the ancestral Wuhan strain—potently activates TRPV1 signaling, driving its redistribution from the nucleus to the cytosol and plasma membrane.

### Single-nucleus RNA sequencing reveals increased transmembrane transporter activity in sensory neurons following BA.5 virus exposure

**Figure 7:**
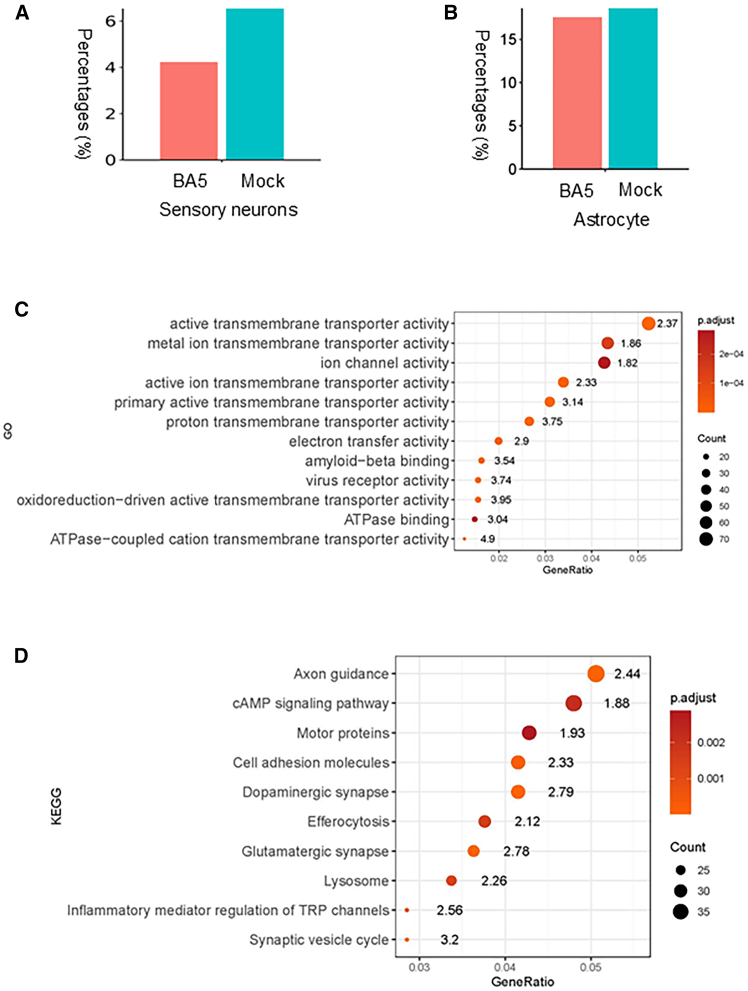
snRNA-seq reveals sensory-neuron responses to BA.5 virus exposure (A) Proportion of sensory neurons decreased following BA.5 infection compared with mock controls. (B) Astrocyte proportions remained relatively unchanged. (C) GO enrichment analysis of sensory neurons showing upregulated molecular functions, including active transmembrane transporter activity and virus receptor activity. Numbers indicate enrichment factors. (D) KEGG pathway enrichment analysis showing altered signaling pathways, including axon guidance, dopaminergic and glutamatergic synapses, cAMP signaling, and inflammatory mediator regulation of TRP channels.

**Figure 8:**
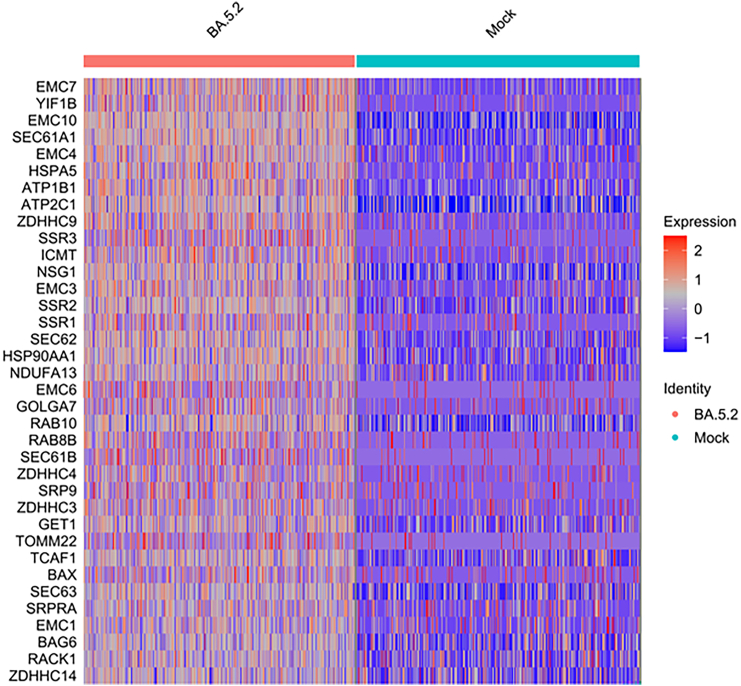
Gene expression heatmap shows upregulation of the exocytosis pathway in response to BA.5 exposure Heatmap of membrane-associated gene expression demonstrating activation of the regulated exocytosis pathway following BA.5 infection, including increased expression of Sec61, EMC, ZDHHC family members, and components of the SSR complex.

To elucidate the molecular effects of SARS-CoV-2 infection on sensory neurons, we performed snRNA-seq analysis of neuronal cultures infected with the Omicron BA.5 variant and compared them to mock-infected controls. The results revealed a marked reduction in the number of sensory neurons in the infected group relative to controls (Figure 7A), whereas glial populations such as astrocytes were less affected (Figure 7B). Gene ontology (GO) enrichment analysis of the sensory neuron subset demonstrated significant upregulation of molecular functions associated with active transmembrane transporter activity and virus receptor activity (Figure 7C). Kyoto encyclopedia of genes and genomes (KEGG) enrichment analysis further revealed that viral exposure significantly altered multiple pathways involved in axon guidance, dopaminergic and glutamatergic synapses, the cAMP signaling pathway, and inflammatory mediator regulation of TRP channels (Figure 7D). A heatmap of genes encoding membrane-localized proteins highlighted upregulation of the regulated exocytosis pathway, including components of the endoplasmic reticulum (ER) primary translocon complex Sec61, the ER membrane protein complex (EMC), palmitoyl acyltransferases (ZDHHC family), and the signal sequence receptor (SSR) complex, all of which facilitate protein trafficking to the plasma membrane (Figure 8).

### BA.5 virus exposure induced axonal degeneration, and the TRPV1 antagonist capsazepine exhibits neuroprotective effects

**Figure 9:**
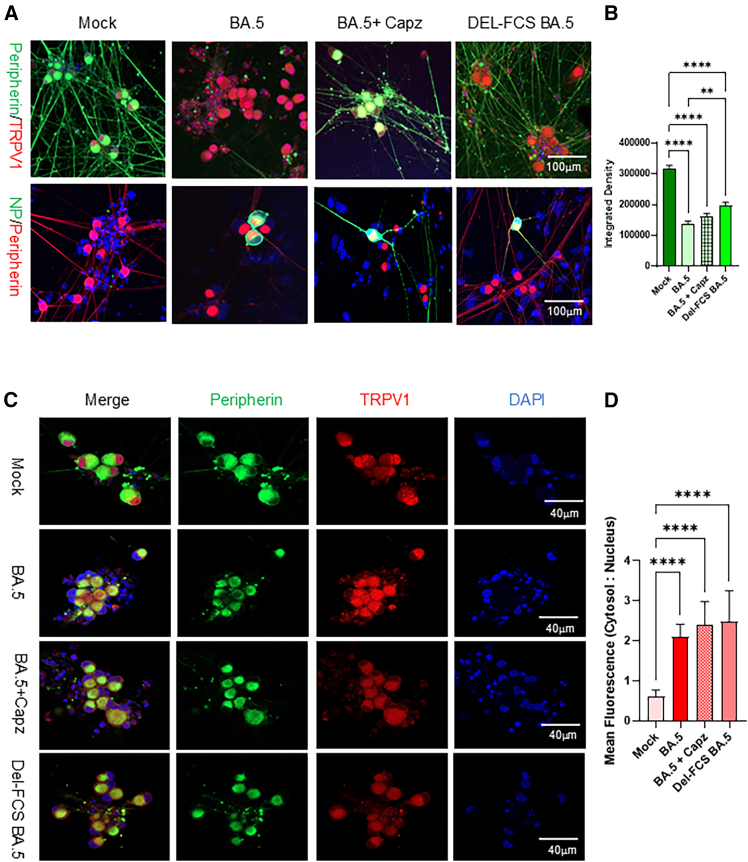
TRPV1 antagonist capsazepine and mutant BA.5 virus alleviate virus-induced axonal injury (A) Immunostaining of neuronal networks exposed to wild-type BA.5, Del-FCS BA.5 mutant, or BA.5 with 10 ng mL^−1^ capsazepine. Cells were stained for peripherin (green), TRPV1 (red), NP (green), and DAPI (blue). BA.5 exposure led to loss of peripherin-positive axons and dendrites, while capsazepine or Del-FCS mutant virus mitigated neuronal damage. Images were obtained using a confocal microscope with a 20× objective. Scale bars, 100 μm. (B) Peripherin fluorescence intensity was quantified in five neuron clusters (each field including cell body and processes) following the exposure to BA.5 with or without capsazepine or Del-FCS mutant virus. Data are presented as mean ± SEM and analyzed using student’s *t* test in GraphPad Prism 10. ∗∗*p* < 0.01, ∗∗∗∗*p* < 0.0001. (C) Immunostaining of neurons under the same conditions with antibodies for TRPV1 (red) and peripherin (green), showing reduced TRPV1 activation following inhibitor or mutant virus exposure. Images were captured with a 40× objective. Scale bars, 20 μm. (D) TRPV1 fluorescence intensity and cytosol-to-nucleus subcellular distribution were quantified in five neurons per field across three independent experiments following. Data represent mean ± SD, analyzed by student’s *t* test using GraphPad Prism 10. ∗∗∗∗*p* < 0.0001, ∗∗∗*p* < 0.001, ∗∗*p* < 0.01, ∗*p* < 0.05.

To further examine the relationship between TRPV1 activation and neuronal degeneration, sensory neurons were co-exposed to the BA.5 virus and the TRPV1 antagonist capsazepine (10 ng mL^−1^). At 48 hpi, neuronal networks were immunostained for peripherin, viral NP, and TRPV1, and imaged at 20× magnification to evaluate axonal morphology. In cultures infected with the BA.5 virus at an MOI of 1.0, peripherin staining revealed pronounced loss of axons and dendrites, indicating neuronal degeneration. Co-administration of capsazepine markedly attenuated this degeneration, preserving axonal integrity. Consistently, NP staining showed that neurons infected in the presence of the TRPV1 inhibitor retained well-defined neuritic processes (Figure 9A).

To assess the contribution of viral entry and replication to TRPV1 activation, we next examined neuronal responses to an attenuated Del-FCS BA.5 mutant strain. This mutant carries a deletion at the S1/S2 furin cleavage site (FCS: KSPRRARSVA) within the SYQTQTKSPRRARSVASQSIIA region, which impairs spike protein cleavage and reduces viral infectivity (unpublished data). Compared with the wild-type BA.5 virus, the Del-FCS mutant elicited markedly lower TRPV1 expression, reduced channel trafficking, and less disruption of neuronal processes (Figures 9A and 9B). Together, these findings indicate that SARS-CoV-2 entry and replication are closely linked to TRPV1 activation, which contributes to axonal damage in sensory neurons. Pharmacological inhibition of TRPV1 with capsazepine provides neuroprotection against BA.5-induced degeneration (Figures 9C and 9D). These results suggest that TRPV1 activation serves as a potential biomarker of SARS-CoV-2-induced neurotoxicity and may represent a viable therapeutic target for mitigating neuronal injury across SARS-CoV-2 variants.

## Discussion

Infection with SARS-CoV-2 is well recognized to cause dysfunction across multiple organ systems, including the nervous system. During both the acute and post-acute phases of infection, in addition to respiratory manifestations, many patients develop neurological symptoms such as headache, anosmia, and ageusia.^2^ Despite extensive research, the precise mechanisms of SARS-CoV-2-induced neurological injury remain incompletely understood. Specifically, it is unclear whether these effects are mediated primarily by direct viral invasion of neurons or by indirect systemic mechanisms such as inflammation, hypoxemia, or coagulopathy.

Two main mechanisms have been proposed to explain how viral infection affects OSNs. The direct mechanism suggests that viruses such as influenza and coronaviruses can infect olfactory receptor neurons and their progenitors directly, leading to neuronal death and loss of olfactory function.^14^ In contrast, the indirect mechanism posits that viruses infect non-neuronal cells within the OE, disrupting epithelial integrity and secondarily impairing underlying neurons.^10^^,^^13^^,^^35^ Our findings, based on both *in vitro* and *in vivo* models, demonstrate that SARS-CoV-2 can directly infect olfactory neurons as well as their basal stem cells. This establishes OSNs and their progenitors as peripheral neural targets of SARS-CoV-2 infection. Moreover, exposure to either the live virus or its S1 spike protein markedly increased ACE2 expression in these neurons, suggesting that recurrent or sustained exposure may potentiate neuronal susceptibility.

The OE is a neuroepithelial tissue uniquely capable of lifelong regeneration, maintained by basal stem cells that replenish olfactory neurons and sustentacular cells after injury. While prior studies focused primarily on OSN and sustentacular cell damage, our hamster model revealed that SARS-CoV-2 infection also disrupts basal stem-cell populations and alters EBF3 expression. EBF3 is strongly expressed during both embryonic and adult neurogenesis and is essential for olfactory neuron differentiation. Because basal progenitors sustain epithelial regeneration, viral damage to these cells likely impairs the regenerative axis, contributing to chronic anosmia—one of the most persistent symptoms of post-viral olfactory dysfunction and long COVID. Further studies should define how SARS-CoV-2 alters basal-cell self-renewal and differentiation programs and evaluate strategies to restore regenerative capacity.

TRPV1-positive sensory neurons, including OSNs, exhibited robust TRPV1 upregulation following Omicron BA.5 infection. Notably, TRPV1 was translocated from the nucleus to the cytosol and inserted into the plasma membrane, even in neurons without detectable viral antigen. TRPV1’s N-terminal ankyrin repeat domains (ARDs) interact with regulatory proteins that control channel gating and trafficking. The SARS-CoV-2 spike protein contains ankyrin-binding motifs-one within the S1 subunit (ACE2 binding) and another in the S2 subunit (membrane fusion), which share structural similarity with TRPV1 ARDs, suggesting potential spike-TRPV1 interactions. Consistent with this hypothesis, exposure of sensory neurons to the BA.5 S1 protein alone was sufficient to induce TRPV1 translocation and activation.

TRPV1-mediated neuronal injury has profound mechanistic and translational implications. Activation of TRPV1 disrupts microtubule organization, causes rapid cytoskeletal disassembly, and impairs axonal transport and growth—processes essential for neuronal connectivity and regeneration. These disruptions likely contribute to sensory neuron loss and cognitive dysfunction observed in post-COVID conditions.^21^ In addition, TRPV1 activation may amplify neuroinflammation through Ca^2+^-dependent release of substance P and calcitonin gene-related peptide, promoting cytokine cascades that further damage the OE and basal-cell niche.

Importantly, our findings that TRPV1 antagonists confer neuroprotection support the hypothesis that TRPV1 activation is a key mediator of SARS-CoV-2-induced neuronal injury. Given that TRPV1 antagonists such as capsazepine and its analogs are already pharmacologically characterized, they may be rapidly repurposed for therapeutic evaluation in post-COVID olfactory dysfunction. Collectively, our results define a mechanistic framework in which SARS-CoV-2 engages TRPV1 signaling to disrupt neuronal integrity and regeneration, offering both a biomarker and a tractable therapeutic target for restoring sensory function after viral infection.

### Limitations of the study

The limitations of our study are that we have not identified how the mechanisms of virus replicated within the neurons and its impact on OSNs regeneration. Ongoing single cell analysis with an EBF3 and TRPV1 knockout cell line would provide a better understanding of the mechanisms of the impact in virus infection.

## Resource availability

### Lead contact

Further information and requests for resources and reagents should be directed to and will be fulfilled by the lead contact, Yiling Hong (yhong@westernu.edu).

### Materials availability

There are no new materials generated in this study.

### Data and code availability

• Single-cell RNA-seq data have been deposited at NCBI BioSample database and are publicly available as of the date of publication. The accession code is SAMN45884840, with the sample name SQ24076636-cellmix-cellmix is listed in the key resources table. Microscopy data reported in this study will be shared by the lead contact upon request.
• This study did not generate custom computational code. The SARS-CoV-2 Omicron BA.5 GISAID accession number: EPI_ISL_13777658 and SARS-CoV-2 BA.2 GISAID accession number: EPI_ISL_9845731 were listed in the key resources table.
• Any additional information required to reanalyze the data reported in this study is available from the lead contact upon request.

## Acknowledgments

This work is supported by the InnoHK-Health program, Innovation Technology Commission, Hong Kong SAR, PR China. The authors would like to thank Libby Liao and Dr. Ma Sidi from Accuramed Technology (Guangzhou, China) Limited for their invaluable assistance with snRNA-seq bioinformatics analysis.

## Author contributions

C.V.S. performed most of the experiments except for those involving the use of live virus exposure, curated the data, and prepared the manuscript. L.S. generated mutant virus, conducted live virus exposure experiments. T.R.C.-Y. conducted live virus exposure experiments. M.B.W.-Y. conducted live virus exposure experiments. L.A.H.-C. performed animal experiments and prepared animal tissue samples for immunofluorescence. C.H. provided funding and resource support, conceptualized the study, and edited the manuscript. H.Y. conceptualized the study, designed methodology, supervised the research, and prepared the manuscript.

## Declaration of interests

The authors declare no competing interests.

## STAR★Methods

### Key resources table

**Table:** 

REAGENT or RESOURCE	SOURCE	IDENTIFIER
**Antibodies**		

Mouse ACE2 Antibody	abcam	Cat# ab89111; RRID: AB_2040508
Rabbit ACE2 Antibody	Invitrogen	Cat# MA5-32307; RRID: AB_2809589
Rabbit EBF Antibody	Invitrogen	Cat# PA5-30985; RRID: AB_2548459
Mouse OMP Antibody	Santa Cruz Biotechnology	Cat# sc-365818; RRID: AB_10842164
Goat OMP Antibody	Fujifilm	Cat# 019-22291; RRID: AB_3094987
Mouse Peripherin Antibody	Invitrogen	Cat# MA3-16724; RRID: AB_568683
Rabbit Peripherin Antibody	abcam	Cat# ab246502
Mouse TMPRSS2 Antibody	Santa Cruz Biotechnology	Cat# sc-515727; RRID: AB_2892118
Rabbit TRPV1 Antibody	abcam	Cat# ab305299; RRID: AB_3105792
Guinea Pig TRPV1 Antibody	Alomone Labs	Cat# ACC-030-GP; RRID: AB_2721813
Rabbit SARS-CoV-2 Spike Glycoprotein S1 Antibody	abcam	Cat# ab283942
Mouse SARS-CoV-2 Nucleocapsid Protein Antibody	Sino Biological	Cat# 40143-MM05; RRID: AB_2827977
Mouse SARS-CoV-2 Nucleocapsid Protein Antibody	Invitrogen	Cat# MA5-29981; RRID: AB_2785780
Rabbit SARS-CoV-2 Nucleocapsid Protein Antibody	Invitrogen	Cat# PA1-41098; RRID: AB_1087200
Goat anti-Rabbit IgG (H + L) Cross-Adsorbed Secondary Antibody, Alexa Fluor™ 546	Invitrogen	Cat# A11010; RRID: AB_2534077
Goat Anti-Mouse IgG H&L (Alexa Fluor® 488)	abcam	Cat# ab150117; RRID: AB_2688012
Goat Anti-Guinea pig IgG H&L (Alexa Fluor® 488)	abcam	Cat# ab150185; RRID: AB_2736871
Goat Anti-Mouse IgG H&L (Alexa Fluor® 647)	abcam	Cat# ab150115; RRID: AB_2687948
Donkey Anti-Rabbit IgG H&L (Alexa Fluor® 647)	abcam	Cat# ab150075; RRID: AB_2752244
Donkey Anti-Goat IgG H&L (Alexa Fluor® 488)	abcam	Cat# ab150129; RRID: AB_2687506
Donkey Anti-Goat IgG H&L (Alexa Fluor® 647)	abcam	Cat# ab150135; RRID: AB_2687955

**Bacterial and virus strains**		

SARS-CoV-2 BA.5	Isolated from COVID-19 patient in Hong Kong	GISAID name: hCoV-19/Hong Kong/HKU-220712-005/2022 GISAID accession number: EPI_ISL_13777658
SARS-CoV-2 BA.5 (with mCherry label)	This paper	NA
SARS-CoV-2 BA.5 (Del-FCS)	This paper	NA
SARS-CoV-2 BA.2	Isolated from COVID-19 patient in Hong Kong	GISAID accession number: EPI_ISL_9845731

**Chemicals, peptides, and recombinant proteins**		

Recombinant Human Coronavirus SARS-CoV-2 Spike Glycoprotein S1 (Active)	Abcam	Cat# ab273068
SARS-CoV-2 Spike S1 Protein, His Tag (BA.4 & BA.5/Omicron)	Acrobiosystems	Cat# S1N-C52Hy
Capsazepine	MedChemExpress	Cat# HY-15640

**Critical commercial assays**		

Chromium Single Cell Fixed RNA Sample Preparation Kit (16 rxns)	10x Genomics	Cat# 1000414

**Deposited data**		

Single Cell Gene Expression Flex of mock- and BA.5-infected iPSC-derived sensory neurons	This paper	BioSample accession: SAMN45884840

**Experimental models: Cell lines**		

BIONi10-C (iPSC)	Sigma-Aldrich	Cat# 66540023
HEK293T	ATCC	CRL-3216; RRID:CVCL_0063
Vero E6	ATCC	CRL-1586; RRID:CVCL_0574

**Experimental models: Organisms/strains**		

Golden Syrian hamsters	Center for Comparative Medicine Research, HKU	HsdHan®:AURA

**Oligonucleotides**		

Primers for FCS-deletion (see Table S1)	This paper	NA
F2A linker and mCherry fragment (see supplemental information)	This paper	NA

**Software and algorithms**		

ImageJ	NA	url: https://imagej.net/ij/
GraphPad Prism 10	NA	url: https://www.graphpad.com/
ZEN Microscopy Software	Zeiss	NA

### Experimental model and study participant details

#### iPSC cells

The human iPSC line BIONi010-C (Sigma-Aldrich, Cat. No. 66540666), distributed by Sigma-Aldrich, has been authenticated. Key authentication and characterization details include the availability of a 16-allele short tandem repeat (STR) fingerprinting profile. The line has been characterized by G-banding for karyotype analysis and is confirmed to possess an APOE3/E3 genotype (or APOE3/4, depending on the specific subclone, as it serves as a parental line for isogenic sets). These lines are officially registered in the hPSCreg database and are commercially available through Sigma-Aldrich. In addition, the cells are tested monthly for mycoplasma contamination to ensure they remain mycoplasma-free.

#### Virus strain

The BA.2 (GISAID accession number: EPI_ISL_9845731) and BA.5 strain (hCoV-19/Hong Kong/HKU-220712-005/2022; GISAID accession number: EPI_ISL_13777658) were isolated from combined nasopharyngeal-throat swabs of COVID-19 patients in Hong Kong. The viral isolate was propagated in VeroE6 cells. When cytopathic effects (CPE) were observed in 80–90% of the cells, the spent culture medium was centrifuged at 1000 × g for 10 min at 4°C. The supernatant was collected, aliquoted, and stored at −80°C as a viral stock. The titer of the viral stock was determined using a plaque assay. All experiments involving authentic SARS-CoV-2 adhered to approved standard operating procedures within the Biosafety Level 3 (BSL-3) facility located at the Department of Microbiology, HKU.

#### Syrian hamster animal model

Male and female Golden Syrian hamsters (strain: HsdHan:AURA; 6–8 weeks old) were obtained from the Center for Comparative Medicine Research, The University of Hong Kong (HKU). All experiments involving live viruses and animal studies were conducted in the HKU Biosafety Level 3 (BSL-3) facility in accordance with approved standard operating procedures. All animal research complied with relevant regulations and ethical guidelines, The study was conducted under the oversight of the Institutional Animal Care and Use Committee (IACUC) at HKU, with species-appropriate care provided and all efforts made to minimize suffering and justify the scientific necessity of the work. The protocol was approved by the Committee on the Use of Live Animals in Teaching and Research, HKU (CULATR-5370-20).

### Method details

#### Generation of peripheral sensory neurons from iPSC-derived

The human iPSC line BIONi010-C (Sigma-Aldrich, Cat# 66540666) was cultured in mTeSR Plus serum-free stem cell culture medium (Stem Cell Technologies, Cat# 85850) at 37°C and 5% CO2. Neural crest cells (NCCs) were generated from iPSCs using Neural Crest Induction Medium (Stem Cell Technologies, Cat# 08610) according to the manufacturer’s instructions. NCCs were further subcultured at a cell density of 2 × 10^5^ cells/cm^2^ in 4-well culture slides and 24-well plates coated in Matrigel gel (Corning, Cat# 354277) for sensory neuron differentiation and maturation using the STEMdiff Sensory Neuron Differentiation Kit (Stem Cell Technologies, Cat# 100–0341) and the STEMdiff Sensory Neuron Maturation Kit (Stem Cell Technologies, Cat# 100–0684) respectively. Mature sensory neurons were maintained in culture for up to 4 weeks. All cells were tested for mycoplasma contamination monthly to ensure they are mycoplasma-free.

#### Generation of recombinant SARS-CoV-2 by circular polymerase extension reaction (CPER)

Viral RNA was extracted from the BA.5 strain and used as a template for cDNA synthesis using Superscript IV Reverse Transcriptase with random hexamer primers according to the manufacturer’s protocol (Invitrogen, Cat# 18090050). Six fragments covering the SARS-CoV-2 genome (F1-F6) with complementary 30-nucleotide ends were amplified from viral cDNA using high-fidelity PrimeSTAR GXL DNA polymerase (Takara Bio, Cat# R050B) with corresponding pairs of primers (Supplementary Table). Equimolar amounts (0.1 pmol each) of the resulting six fragments and SARS-CoV-2 linker fragments were assembled into a circular full-length cDNA using PrimeStar GXL DNA polymerase under the following cycling conditions: initial denaturation at 98°C for 1 min, followed by 35 cycles of denaturation at 98°C for 10 s, annealing at 60°C for 10 s, and extension at 68°C for 15 min, followed by a final extension at 68°C for 15 min.

#### Generation of mCherry-expressing BA.5 virus

To generate the BA.5-mCherry virus, we inserted the F2A linker (Table S2) and mCherry fragment into the BA.5 virus sequence between the orf7a and orf7b regions. The resulting CPER product was transfected into HEK293T cells using Lipofectamine LTX with Plus Reagent (Invitrogen, Cat# 15338100), following the manufacturer’s protocol. Six hours post-transfection, the cells were trypsinized and transferred to a confluent monolayer of Vero E6 cells in a T25 flask for virus propagation to generate the viral stock.

#### Generation of Del-FCS BA.5 virus

To generate Del-FCS BA.5 virus, the sequence for the furin cleavage site (FCS) located in BA.5 fragment F5 was deleted through CPER technique. The F5 fragment with the deleted FCS was assembled with BA.5 F1-F4 and F6 fragments using CPER. The resulting CPER product was transfected into HEK293T cells using Lipofectamine LTX with Plus Reagent, following the manufacturer’s protocol. At 6 h post-transfection, the cells were trypsinized and transferred to a confluent monolayer of Vero E6 cells in a T25 flask for virus propagation to generate the viral stock.

#### Exposure of sensory neuronal networks to virus and its S1 protein

BA.5 and Wuhan spike protein S1 subunit (abcam, Cat# ab273068, and Acrobiosystems, Cat# S1N-C52Hy) were diluted in Sensory Neuron Maturation Medium to 100 ng/ml. Neural crest cells, seeded at a density of 2 × 10^5^ cells/cm^2^, were cultured in 4-well culture slides and 24-well plates coated with Matrigel. Following neuronal differentiation and maturation, the peripheral sensory neurons were exposed to SARS-CoV-2 BA.5, mCherry-expressing BA.5, or Del-FCS BA.5 virus diluted in Sensory Neuron Maturation Medium (from the STEMdiff Sensory Neuron Maturation Kit) at MOIs of 0.1, 1, or 2. An MOI of 1 corresponds to a 1:1 ratio of cells to virus particles. Mock-exposed cultured neurons served as controls. Capsazepine (MedChemExpress, Cat# HY-15640) at 10 μM was included in some BA.5-exposed cultures for TRPV1 inhibition studies. Cultures were incubated at 37°C with 5% CO2 for 24 or 48 h. After incubation, the supernatant was removed, and the cells were washed with PBS prior to being processed for immunofluorescence staining. Indicate here the number of replicates for each condition.

#### Viral challenge of syrian hamsters

The animal infection experiments were performed as described previously.^36^ Male and female Golden Syrian hamsters (strain: HsdHan:AURA) (6–8 weeks old) were obtained from the Center for Comparative Medicine Research, HKU. A challenge dose of 1x105 plaque-forming units BA.2 in 100 μL Dulbecco’s modified Eagle’s Media (DMEM) was intranasally inoculated to each hamster through under intraperitoneal ketamine (200 mg) and xylazine (10 mg/kg) anesthesia. All animals will be transferred to a BSL-3 animal facility for the virus challenge. The hamsters were sacrificed for histological study at 4 days post inoculation (dpi.). All experiments involving live viruses and animal studies were conducted at HKU BSL-3 facility following the approved standard operating procedures. The protocol has been approved by the Committee on the Use of Live Animals in Teaching and Research (CULATR-5370-20).

#### Immunofluorescence (IF) staining and microscopy

Neurons were fixed with 4% paraformaldehyde in PBS for 15 min, washed with PBS, and permeabilized with 0.1% Triton X- in PBS for 10 min. The neurons were blocked in 1% BSA for 1 h at room temperature and incubated with primary antibodies against peripherin (Invitrogen, Cat# MA3-16724, and abcam, Cat# ab246502), TRPV1 (Abcam, Cat #ab305299), two ACE2 (Invitrogen, Cat #MA5-32307, and abcam, Cat# ab89111), OMP (Santa Cruz Biotechnology, Cat #sc390543,SARS-CoV-2 S1 subunit (abcam, Cat# ab283942), SARS-CoV-2 nucleocapsid protein (Sino Biological, Cat# 40143-MM05) separately or in various combinations, overnight at 4°C. After primary antibody incubation, the cells were washed and incubated with secondary antibodies for 1 h at room temperature. Coverslips were mounted with VECTASHIELD PLUS Antifade Mounting Medium with DAPI (Vector Laboratories, Cat #H-1200-10).

Skulls of the hamsters were harvested, fixed and processed into 4 μm sagittal sections as previously described.^37^ The tissues were deparaffinized, rehydrated and unmasked with Citrate-based Antigen Unmasking Solution (Vector Laboratories, Cat #H-3300-250) at 60°C overnight. The tissues were incubated in Sudan Black B (Sigma-Aldrich, Cat #199664) for 15 min and blocked in 1% BSA for 1 h at room temperature and incubated with primary antibodies against TRPV1 (Abcam, Cat #ab305299), EBF3 (Invitrogen Cat #PA5-30985), OMP (Fujifilm, Cat #019–22291) and SARS-CoV-2 nucleocapsid protein (Invitrogen, Cat #MA5-29981 and Cat #PA1-41098) at 4°C overnight. The next day, the cells were washed and incubated with secondary antibodies for 1 h at room temperature and mounted with VECTSHIELD PLUS Antifade Mounting Medium with DAPI (Vector Laboratories, Cat #H-1200-10).

Phase contrast images were captured using EVOS M5000 Imaging System (Thermo Fisher Scientific, USA). Immunostained images were captured using a Zeiss LSM 880 confocal microscope. Representative images for each experimental condition were chosen for inclusion in figures. For expression analysis data were collected from three independent images and analyzed using ImageJ software (https://imagej.nih.gov/ij/).

#### Immunofluorescence analysis

ImageJ was used to quantify cell fluorescence after IF. Three biological replicates per condition were taken for each experimental group, and three to five neurons were selected from each image for quantification.

For ACE2 quantification, the cell body is cropped and measured for the mean fluorescence in the cropped area. The average mean fluorescence of the mock-exposed cells were used as a benchmark to calculate the fold change of mean fluorescence of BA.5-infected cells or S1 glycoprotein-exposed cells.

For TRPV1 quantification, the nucleus and cytosol of selected cells were cropped to quantify their integrated density separately to showcase the distribution and expression of TRPV1 within the cell. The ratio of nuclear mean fluorescence to cytosolic mean fluorescence was also calculated to demonstrate the activation of TRPV1 in different treatment groups.

For peripherin quantification, five fields from cells clusters (each field including cell body and processes) were selected for each group, and cropped to measure the integrated density of peripherin expression.

Thickness of EBF+ cells in animal tissues was measured in three areas each from three mock and BA.2- infected group, using ZEN microscopy software (ZEISS). three fields in each group were cropped to measure the integrated density of EBF3 expression. Measured data was subjected to one-way ANOVA and Student’s *t* test; a *p*-value <0.05 was considered significant. Correlations were determined by calculating Pearson coefficients.

#### Single-nuclei RNA sequencing (snRNA-seq) sample preparation

Mock-infected or BA.5-infected mature sensory neurons (*n* = 2) were harvested after 24 h. Neurons were incubated in Accutase (Stem Cell Technologies, Cat #07920) at 37°C for 7 min and detached cells were centrifuged at 300 xg for 5 min. The cell pellet was resuspended in 200 μL of pre-chilled Lysis Buffer (10 mM Tris-HCl, pH 7.4, 10 mM NaCl, 3 mM MgCl, 0.025% Nonidet P40 Substitute in PBS), followed by a 1-min incubation on ice. Next, 800 μL of pre-chilled Nuclei Wash Buffer (1% BSA, 0.2 U/μL RNase Inhibitor in PBS) was added to the lysate, and the mixture was centrifuged at 500 xg for 10 min. The resulting nuclei pellet was washed with 1 mL of Nuclei Wash Buffer, centrifuged again, and resuspended in 1 mL of the same buffer. The nuclei were then immediately fixed using the Chromium Next GEM Single Cell Fixed RNA Sample Preparation Kit (10X Genomics, Cat #1000414), following the manufacturer’s instructions. Fixed nuclei samples were intermittently evaluated for count and quality using the LUNA dual fluorescence cell counter (Logos Biosystems).

The fixed nuclei samples were sequenced using the Single Cell Gene Expression Flex for Multiplexed Samples (10X Genomics) protocol. Following Probe Hybridization, uniquely barcoded nuclei were counted and pooled in equal numbers, according to manufactuer’s protocol. The pooled nuclei were washed, targeting 20,000 cells per sample for partitioning into GEMs on Chip Q, GEM barcoding, GEM recovery, pre-amplification, and library construction. Sequencing libraries were prepared in accordance with the 10X Genomics User Guide (CG000527). Libraries were sequenced on an Illumina NovaSeqX platform using paired-end dual-indexing with a read depth of approximately 300 million reads per sample and a 2 × 150 read length. Demultiplexing of sequencing libraries was performed with bcl2fastq (Illumina), and FASTQ files were processed using Cell Ranger v7.1.0 (10X Genomics) with the GRCh38-2020-A reference genome.

#### snRNA-seq data analysis

##### Quality control of scRNA-seq data

Raw reads were aligned to the human genome (hg38), and cells were identified using the *Cell Ranger count* pipeline (v7.1.0). Cells with fewer than 200 detected genes, more than 8,000 detected genes, or a mitochondrial contamination rate exceeding 10% were filtered out as low-quality cells. To further reduce ambient RNA contamination from droplets, the *SoupX* R package (v1.6.1) was applied with default settings. Additionally, potential doublets were removed using the *DoubletFinder* R package (v2.0.3) with default parameters. Quality control and normalization of the scRNA-seq data were subsequently performed using the *Seurat* R package (v4.3.0).

#### Cell clustering and cell-type annotation

The gene expression matrices from the filtered cells were normalized to the total UMI counts per cell and transformed to the natural log scale. Highly variable genes (HVGs) were identified using the *FindVariableFeatures* function, selecting the top 2000 HVGs from the corrected expression matrix. The data were then centered and scaled after regressing out cell cycle effects (S and G2M scores were calculated using the *CellCycleScoring* function in *Seurat*). Principal component analysis (PCA) was performed on the HVGs using the *RunPCA* function. To correct for batch effects, the *RunHarmony* function was applied with default parameters.

Dimensionality reduction and k-nearest neighbor graph construction (k = 20) were conducted using the *FindNeighbors* function, based on Euclidean distances in the 50-dimensional PC space. Cell clusters were identified using the Louvain-Jaccard graph-based method, with the clustering resolution set to 0.2 via the *FindClusters* function. For visualization, the *RunUMAP* function was employed to reduce high-dimensional data into a two-dimensional (2D) representation using dimensions 1–20.

Cluster-specific marker genes were identified using the *FindAllMarkers* function with default parameters, and significance in gene expression differences was determined by the Wilcoxon rank-sum test with Bonferroni correction. Cell types were manually annotated based on cluster markers. To determine sample composition by cell type, the number of cells for each type in each sample was calculated, normalized to the total number of cells per sample, and scaled to 100% for each cell type.

#### Cell-type subclustering

For the major cell types, such as astrocytes and neurons, we repeated the previously described steps (normalization, dimensionality reduction, and clustering) to identify subclusters. These subclusters were subsequently annotated as distinct specific cell subtypes. To determine the genes uniquely expressed in each subcluster, we utilized the *FindAllMarkers* function in *Seurat* with default parameters. To calculate the composition of different groups within each subcluster, the number of cells from each group within each subcluster was counted and analyzed.

#### GO and KEGG enrichment analysis

Enrichment scores (*p*-values) for selected GO/KEGG annotations were calculated using the *clusterProfiler* R package (v4.2.2) through a hypergeometric statistical test with a significance threshold of 0.05. The Benjamini-Hochberg method was applied to estimate the false discovery rate (FDR). Enrichment analysis was performed on the input gene lists, with all genes listed in the *org.Hs.*e.g.,*.db* database serving as the background. Finally, the dot*plot* function was used for visualization of the results.

### Quantification and statistical analysis

Bar charts are presented as mean ± SEM, calculated using GraphPad Prism 10 (GraphPad Software, Inc.). The bar height represents the mean, and the error bars indicate the SEM. For comparisons of fluorescence intensity, data were tested for normality and analyzed using one-way ANOVA or Student’s *t* test in GraphPad Prism 10 (Analyze function); a *p*-value <0.05 was considered statistically significant. Correlations were assessed using Pearson’s correlation coefficient. Detailed statistical information for each analysis is provided in the corresponding figure legends.

## Footnotes

Supplemental information can be found online at https://doi.org/10.1016/j.isci.2026.116098.
