# Supplementary material for: SARS-CoV-2 infects olfactory neurons and basal stem cells and induces axonal degeneration through TRPV1 activation
Source: iScience. 2026 May 25;29(6):116098. doi: 10.1016/j.isci.2026.116098 (PMC13224126; doi:10.1016/j.isci.2026.116098)
Supplement: Document S1. Figures S1–S4, Tables S1 and S2 [file mmc1.pdf]

iScience, Volume *29*

Supplemental information

SARS-CoV-2 infects olfactory neurons and basal stem cells and induces axonal degeneration through TRPV1 activation

Vanessa Anna Co, Siwen Liu, Rachel Chun-Yee Tam, Bobo Wing-Yee Mok, Alvin Hiu-Chung Lam, Honglin Chen, and Yiling Hong

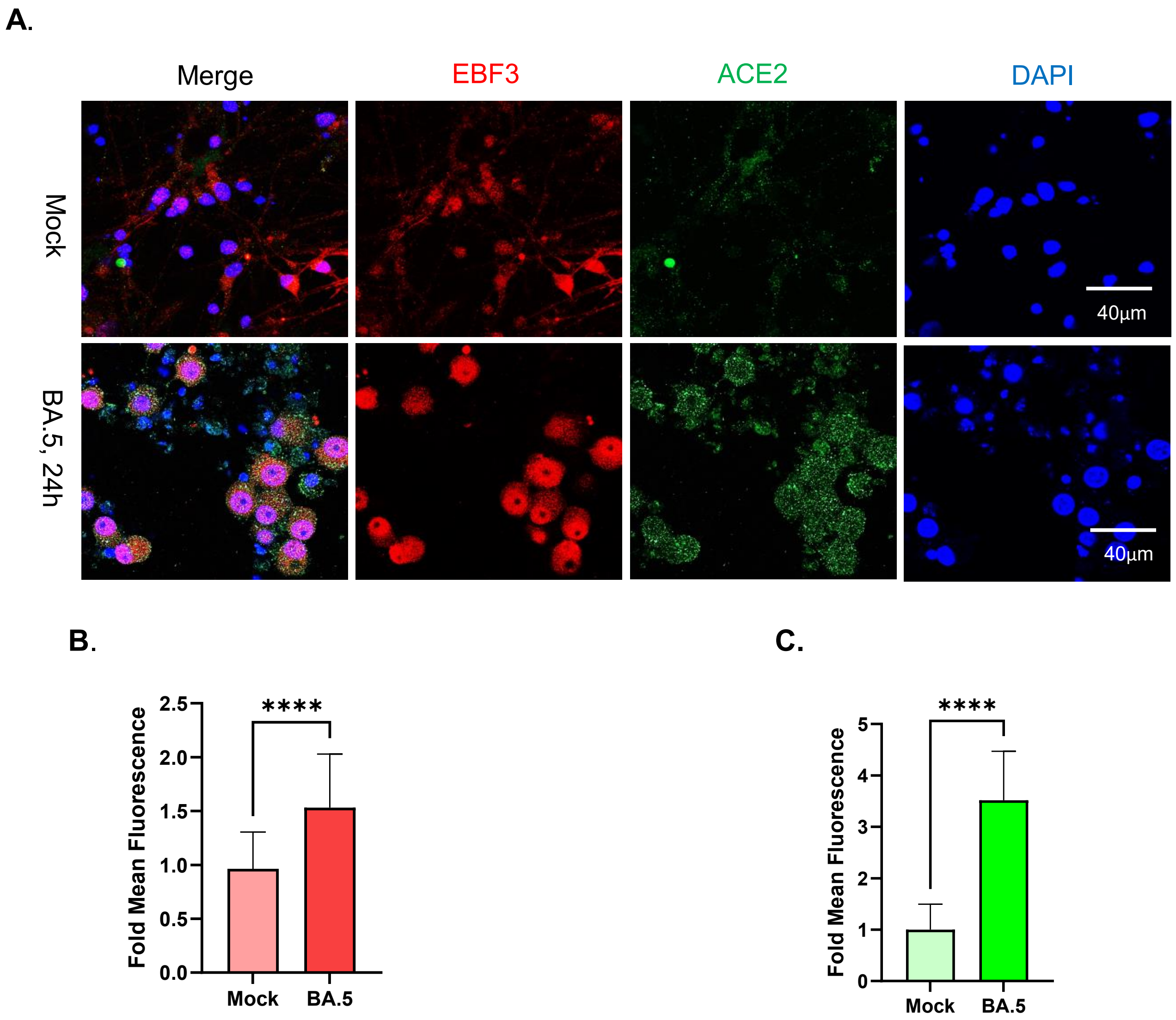

Figure S1. SARS-CoV-2 exposure induces ACE2 expression in EBF3-expressing olfactory neurons. (A) Immunostaining of olfactory neurons labeled with ACE2 (green) and EBF3 (red), with DAPI marking nuclei, showing increased ACE2 expression following exposure to the SARS-CoV-2 BA.5 variant. Images were acquired using a confocal microscope with a 40× objective. Scale bar, 40 μm. (B) Quantification of EBF3 fluorescence intensity using ImageJ in mock- and BA.5-exposed neurons from three independent studies. Data are represented as mean ± SEM, analyzed by Student's *t*-test using GraphPad Prism 10, **** $p < 0.0001$. (C) Quantification of ACE2 fluorescence intensity using ImageJ in mock- and BA.5-exposed neurons from three independent studies. Data are represented as mean ± SEM, analyzed by Student's *t*-test using GraphPad Prism 10, **** $p < 0.0001$.

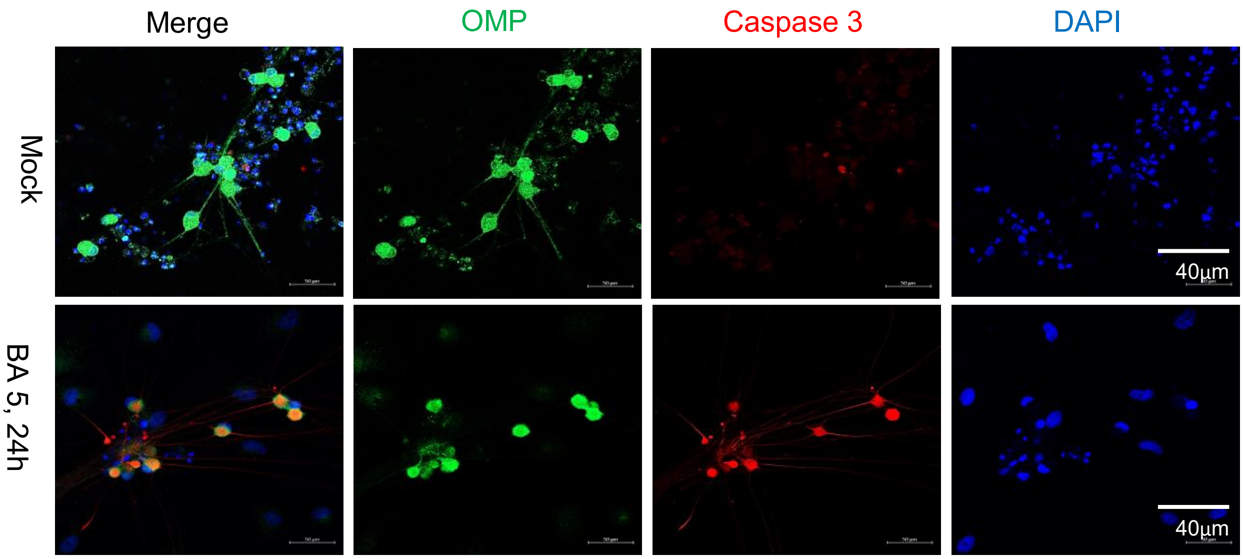

Figure S2. SARS-CoV-2 exposure induces apoptosis in OMP-expressing olfactory sensory neurons. Immunostaining of olfactory sensory neurons labeled with OMP (green) and the apoptotic marker caspase-3 (red), with DAPI marking nuclei, shows increased caspase-3 expression following exposure to the SARS-CoV-2 BA.5 variant. Images were acquired using a confocal microscope with a 40× objective, scale bar, 40 µm.

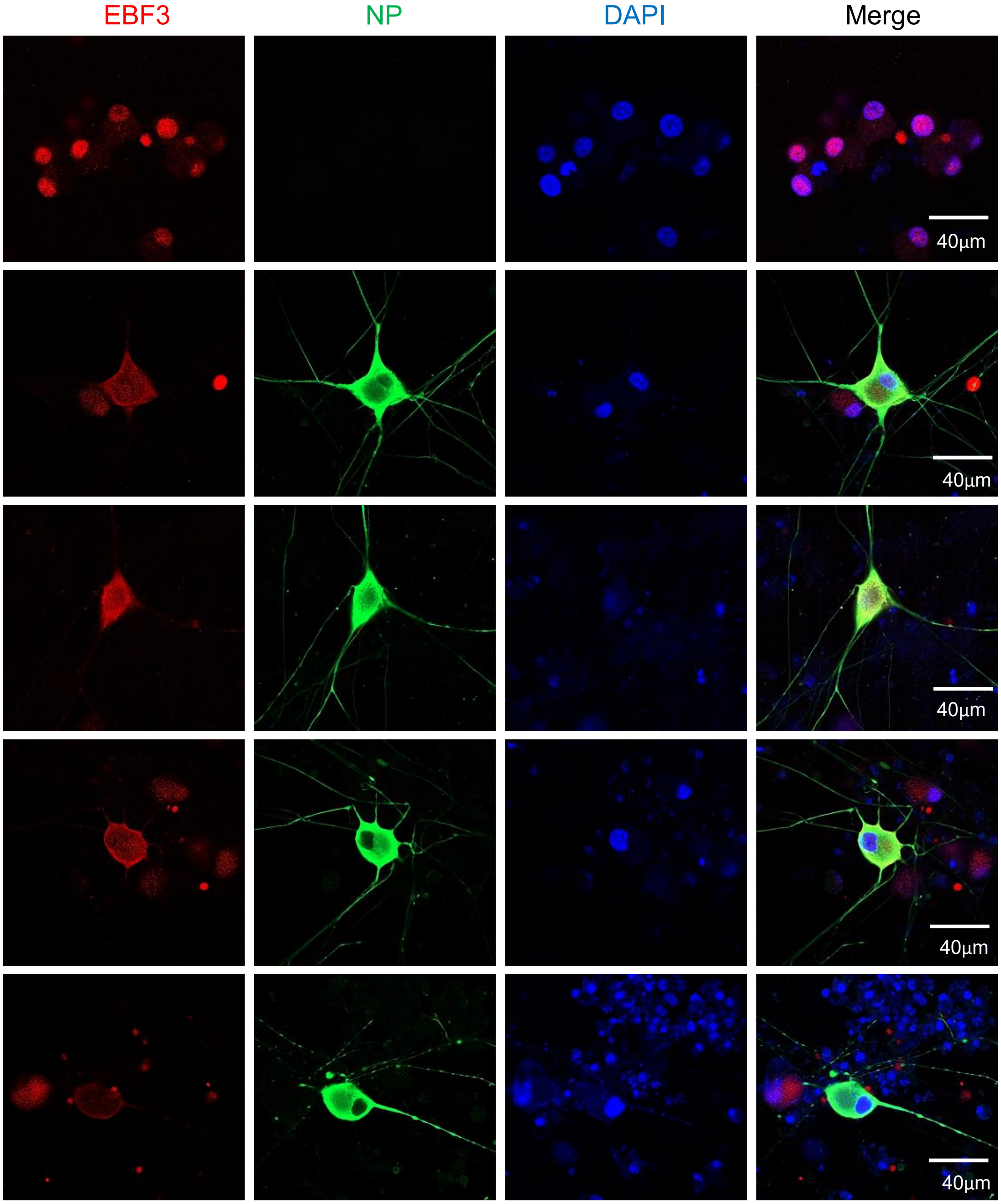

Figure S3. Detection of SARS-CoV-2 nucleocapsid protein in olfactory sensory, mitral, and tufted neurons. Immunostaining of olfactory neuron subtypes for the SARS-CoV-2 Omicron BA.5 nucleocapsid protein (NP, green) together with the neuronal marker EBF3 (red). Images were acquired using a confocal microscope with a 40× objective, scale bar, 40 µm.

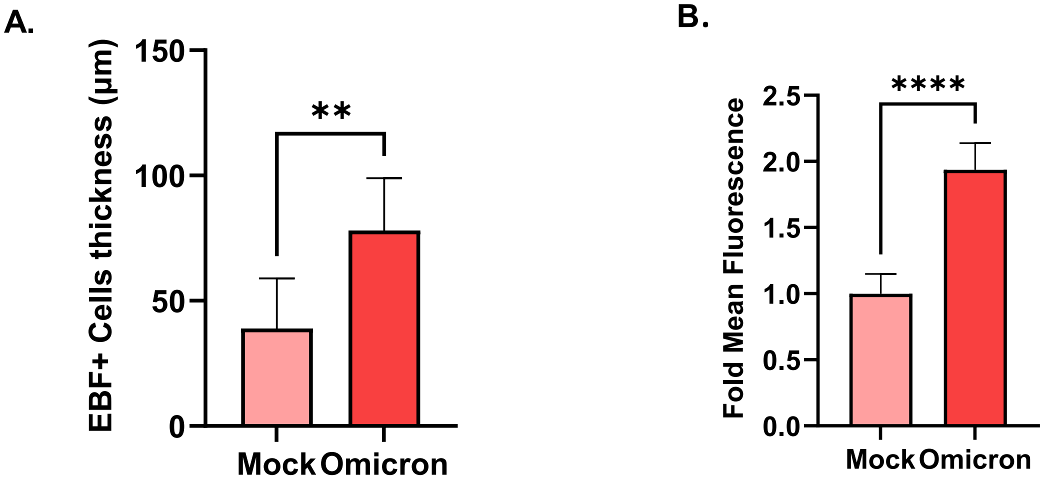

Figure S4. SARS-CoV-2 exposure promotes olfactory basal cell proliferation in the hamster model. (A) Quantification of the thickness of EBF3-expressing cell layers in the hamster olfactory epithelium using ImageJ. Data are represented as mean ± SEM, analyzed by Student's *t*-test using GraphPad Prism 10, $**p < 0.01$. (B) Quantification of EBF3 fluorescence intensity in olfactory epithelium from mock- and BA.5-exposed hamsters using ImageJ from three independent studies. Data are represented as mean ± SEM, analyzed by Student's *t*-test using GraphPad Prism 10, $****\ p < 0.0001$.

Supplementary Table 1. Primer Sequence for Del-FCS BA.5 Generation

	Primer sequence (5' to 3')
NC-F1-V3-F	ATTAAAGGTTTATACCTTCCCAGGTAACAAACCAACCAAC
NC-F1-V3-R	GACATGTCCACAACTTGCGTGTGGAGGTTAATGTTGTCTACTG
NC-F2-V3-F	CAACAGTAGACAACATTAACCTCCACACGCAAGTTGTGGACATGTC
NC-F2-V3-R	CTAACACTGAAAAAGTCTGTCCTGGTTGAATGCGAACAAAC
NC-F3-V3-F	GTATAAGTTTGTTCGCATTCAACCAGGACAGACTTTTTCAGTGTTAG
NC-F3-V3-R	CATATAGTGAACCGCCACACATGACCATTTCACTCAATACTTG
NC-F4-V3-F	GAGTGTGCTCAAGTATTGAGTGAAATGGTCATGTGTGGCGG
NC-F4-V3-R	CCACTGTCTTAAAACAGCTGTACCTGGTGCAACTCCTTTATCAGAACC
NC-F5-V3-F	CCCTATAATATGAGAGTTATACATTTTGGTGCTGGTTCTGATAAAGG
NC-F5-V3-R	CGTACTCATCAGCTTGTGCTTACAAAGGCACGCTAGTAGTCG
NC-F6-V3-F	GTTAATCCAGTAATGGAACCAATTTATGATGAACCGACGACGACTAC
NC-F6-V3-R	GTCATTCTCCTAAGAAGCTATTAAAATCACATGGGG

Supplementary Table 2. F2A Linker and mCherry Fragment Sequence

CTGAATTTTGACCTTCTTAAGCTTGCGGGAGACGTCGAGTCCAACCCTGGGCCTAT
GGTGAGCAAGGGCGAGGAGGATAACATGGCCATCATCAAGGAGTTCATGCGCTTCA
AGGTGCACATGGAGGGCTCCGTGAACGGCCACGAGTTCGAGATCGAGGGCGAGG
GCGAGGGCCGCCCCTACGAGGGCACCCAGACCGCCAAGCTGAAGGTGACCAAGG
GTGGCCCCCTGCCCTTCGCCTGGGACATCCTGTCCCCTCAGTTCATGTACGGCTC
CAAGGCCTACGTGAAGCACCCCGCCGACATCCCCGACTACTTGAAGCTGTCCTTCC
CCGAGGGCTTCAAGTGGGAGCGCGTGATGAACTTCGAGGACGGCGGCGTGGTGA
CCGTGACCCAGGACTCCTCCCTGCAGGACGGCGAGTTCATCTACAAGGTGAAGCT
GCGCGGCACCAACTTCCCCTCCGACGGCCCCGTAATGCAGAAGAAGACCATGGGC
TGGGAGGCCTCCTCCGAGCGGATGTACCCCGAGGACGGCGCCCTGAAGGGCGAG
ATCAAGCAGAGGCTGAAGCTGAAGGACGGCGGCCACTACGACGCTGAGGTCAAGA
CCACCTACAAGGCCAAGAAGCCCGTGCAGCTGCCCGGCGCCTACAACGTCAACAT
CAAGTTGGACATCACCTCCCACAACGAGGACTACACCATCGTGGAACAGTACGAAC
GCGCCGAGGGCCGCCACTCCACCGGCGGCATGGACGAGCTGTACAAGGAATGA
